# Absence of “Warm-Up” during Active Avoidance Learning in a Rat Model of Anxiety Vulnerability: Insights from Computational Modeling

**DOI:** 10.3389/fnbeh.2014.00283

**Published:** 2014-08-18

**Authors:** Catherine E. Myers, Ian M. Smith, Richard J. Servatius, Kevin D. Beck

**Affiliations:** ^1^Department of Veterans Affairs, VA New Jersey Health Care System, East Orange, NJ, USA; ^2^Stress and Motivated Behavior Institute, Department of Neurology and Neurosciences, New Jersey Medical School, Rutgers, The State University of New Jersey, Newark, NJ, USA

**Keywords:** reinforcement learning model, anxiety vulnerability, acquisition, extinction, learning and memory

## Abstract

Avoidance behaviors, in which a learned response causes omission of an upcoming punisher, are a core feature of many psychiatric disorders. While reinforcement learning (RL) models have been widely used to study the development of appetitive behaviors, less attention has been paid to avoidance. Here, we present a RL model of lever-press avoidance learning in Sprague-Dawley (SD) rats and in the inbred Wistar Kyoto (WKY) rat, which has been proposed as a model of anxiety vulnerability. We focus on “warm-up,” transiently decreased avoidance responding at the start of a testing session, which is shown by SD but not WKY rats. We first show that a RL model can correctly simulate key aspects of acquisition, extinction, and warm-up in SD rats; we then show that WKY behavior can be simulated by altering three model parameters, which respectively govern the tendency to explore new behaviors vs. exploit previously reinforced ones, the tendency to repeat previous behaviors regardless of reinforcement, and the learning rate for predicting future outcomes. This suggests that several, dissociable mechanisms may contribute independently to strain differences in behavior. The model predicts that, if the “standard” inter-session interval is shortened from 48 to 24 h, SD rats (but not WKY) will continue to show warm-up; we confirm this prediction in an empirical study with SD and WKY rats. The model further predicts that SD rats will continue to show warm-up with inter-session intervals as short as a few minutes, while WKY rats will not show warm-up, even with inter-session intervals as long as a month. Together, the modeling and empirical data indicate that strain differences in warm-up are qualitative rather than just the result of differential sensitivity to task variables. Understanding the mechanisms that govern expression of warm-up behavior in avoidance may lead to better understanding of pathological avoidance, and potential pathways to modify these processes.

Anxiety disorders are the most common psychiatric disorders, with a worldwide lifetime prevalence of 16–29% (Kessler et al., 2005; Somers et al., 2006). Although each subtype (e.g., generalized anxiety disorder, obsessive-compulsive disorder, panic disorder, and social phobia) has unique features, a core symptom of all anxiety disorders is excessive avoidance. Avoidance is also a defining symptom for posttraumatic stress disorder (PTSD), and the growth of avoidance behaviors traces the full expression of PTSD (North et al., 2004; Karamustafalioglu et al., 2006; O’Donnell et al., 2007; Kashdan et al., 2009). Given this prominent position, acquisition and maintenance of avoidance behaviors may represent an endophenotype for a variety of anxiety- and stress-related mental disorders (Gould and Gottesman, 2006).

Among a variety of neurobiological and neurobehavioral factors representing a source of risk for pathological avoidance, some have been amenable to study in animal models. For example, the personality trait of behavioral inhibition, characterized as extreme withdrawal in the face of social and non-social challenges (Kagan et al., 1987; Rosenbaum et al., 1991; Fox et al., 2005), is consistently linked to anxiety disorders (Kagan et al., 1987; Hirshfeld et al., 1992; Biederman et al., 1993; Rosenbaum et al., 1993; Fox et al., 2005; Hirshfeld-Becker et al., 2007). Behavioral inhibition can be studied via an animal model, the inbred Wistar Kyoto (WKY) rat strain, which displays behavioral withdrawal, propensity to avoid, hyper-responsiveness to stress, and hypervigilance, compared to outbred strains such as the Sprague-Dawley (SD) rat (Pare, 1992, 1993; Solberg et al., 2001; Drolet et al., 2002; McAuley et al., 2009; Lemos et al., 2011). Thus, WKY rats represent an animal model of behavioral withdrawal in the face of social and non-social challenges (Jiao et al., 2011b).

It has therefore been useful to compare the acquisition and maintenance of avoidance behavior in the SD and WKY rat models. For example, in lever-press avoidance, a rat is placed in a conditioning chamber for several acquisition trials; on each trial, a warning signal *W*, such as a tone, is presented for some interval (warning period), and then remains on during a subsequent shock period during which electric shocks are delivered every few seconds. If the animal presses a lever during the shock period, this is defined as an escape response: both *W* and shocks are terminated, and the trial moves immediately to an intertrial interval (ITI). If the animal presses the lever during the warning period, this is defined as an avoidance response: *W* is terminated, no shocks are delivered, and the trial moves immediately to the ITI. Behaviorally inhibited WKY rats acquire avoidance responses more quickly than SD rats (Servatius et al., 2008; Beck et al., 2011; Jiao et al., 2011b; Perrotti et al., 2013). WKY rats also typically show impaired extinction of responding when *W* is no longer paired with shock (Servatius et al., 2008; Beck et al., 2011; Jiao et al., 2011b; Perrotti et al., 2013). This impaired extinction indicates that the WKY rat is an overly avoidant animal that is willing to expend energy and continue displaying the avoidance response during extinction rather than occasionally testing whether the reinforcement contingency is still present. Such resistance to extinction has been implicated in neuropathology of human anxiety (Myers and Davis, 2002; Barad, 2005).

A curious feature that appears across avoidance learning paradigms emerges when one looks at behavior within, rather than across, sessions. Specifically, SD rats typically show less avoidance responding at the start of a daily session, compared to their performance at the end of the prior session or later in the current session (Servatius et al., 2008). This phenomenon has been termed “warm-up,” and is shown by a number of species in a range of avoidance paradigms (for reviews, see Kamin, 1963; Spear et al., 1973; Hineline, 1978). In contrast, WKY rats tend to respond on the first trial of each session at approximately the same rate as at the end of the prior session (Servatius et al., 2008; Perrotti et al., 2013). It is possible that the absence of warm-up contributes to the generally faster acquisition, and slower extinction, of avoidance in the WKY rats compared to SD rats. Thus, understanding the nature of the warm-up phenomenon may have implications for the study of avoidance learning, and may in turn provide insight into how pathological avoidance is acquired and maintained in anxiety-vulnerable humans.

Several general classes of explanation for warm-up have been presented (for review, see McSweeney and Roll, 1993; Beck et al., 2010). Perhaps the simplest explanation invokes simple forgetting of the avoidance response during the inter-session interval, with warm-up reflecting reacquisition during the beginning of the next session. However, simple forgetting does not appear to be an adequate explanation, since warm-up can occur with inter-session intervals as short as 30 min (Hineline, 1978). Another early explanation for warm-up was that the decrement in responding on early trials of a session could be the result of a context shift, as the animal is moved from the home cage into the testing chamber, and these contextual effects need time to dissipate before the animal can begin executing avoidance responses. However, this explanation also appears unlikely since warm-up is not reduced if the animals are given a period of confinement in the experimental chamber before the session begins (Hoffman et al., 1961), nor is warm-up abolished if the animals are housed round-the-clock in the experimental chamber to eliminate context effects (Hineline, 1978).

Another class of explanations for the warm-up effect suggests that it reflects emotional processing. On the one hand, some researchers have suggested that presentation of shocks, early in a testing session, might produce arousal that needs to be overcome before the animal can begin executing avoidance responses (Hoffman and Fleshler, 1962); such arousal might produce a species-specific response such as freezing that could transiently interfere with the animal’s ability to execute a lever-press response. However, this explanation fails to account for the fact that warm-up is relatively unaffected by shock intensity (Hoffman et al., 1961), or for the decrement in responding observed on the very first trial of a session, before any shock has yet been delivered. On the other hand, researchers have suggested that presentation of several shocks may be required before arousal accumulates sufficiently to motivate responding (Hoffman et al., 1961; Powell, 1972). However, this explanation fails to account for the fact that warm-up can be observed even during extinction sessions, when no shocks are presented (e.g., Bullock, 1960; Nakamura and Anderson, 1962). Thus, while emotional effects, including freezing, may certainly occur during and contribute to acquisition and extinction of avoidance, they alone do not appear sufficient to fully account for the phenomenon of warm-up (Nakamura and Anderson, 1962; Spear et al., 1973).

A final class of explanations for the warm-up effects invokes the concept of interference. For example, Spear et al. (1973) conducted a series of studies showing that warm-up could be reduced by pretest treatments that appeared to affect memory of the prior session(s) rather than affecting motivation in the current session. They concluded that an important factor contributing to warm-up was the lingering influence of “unspecified events” occurring between learning and testing, such as the intervention of other behaviors during the inter-session period, which interfered with retrieval of the memory trace for the avoidance response. An interference account of warm-up avoids many of the difficulties inherent in the other explanations, since it presumes interference is possible even with a relatively short inter-session interval, should be relatively independent of shock intensity, and should indeed be maximal on the first trial of a session, even before shock has occurred. On the other hand, the central weakness of this account is that it invokes the influence of hypothetical events that occur during the inter-session interval, when the animal’s behavior is often not observed and may be difficult to qualify much less quantify. Evaluating the nature and impact of such unspecified events has therefore proven understandably difficult in empirical studies, but computational modeling provides a possible tool to approach this issue, and to determine whether such hypothetical interference from prior behaviors could indeed replicate the existing data on warm-up effects.

Many computational models of associative learning exist, often using a reinforcement learning (RL) model which consists of two modules, the actor and the critic (Barto et al., 1983). The critic receives as input the current state, defined as the configuration of external and internal stimuli, and learns to output the “goodness” or reward value of each state. In the absence of explicit reward or punishment, learning can also be driven by changes in the prediction of future reward or punishment (Sutton, 1988; Dayan and Balleine, 2002). The critic sends these prediction values to the actor which learns through trial and error to select from a set of possible responses in order to maximize future reward and minimize future punishment (Dayan and Balleine, 2002). Such models therefore embody aspects of several theories of avoidance learning, including two-factor theory (Mowrer, 1951), which posits separate stimulus–stimulus and stimulus–outcome learning processes, and cognitive expectancy theories, which posit that organisms learn to select among possible responses based on the expected long-term outcome from each (Tolman, 1932; Seligman and Johnston, 1973). Actor–critic models have been widely used by many researchers to understand the roles of brain substrates, such as the nigrostriatal dopamine system, the dorsal striatal action selection system, the prefrontal cortex, and the hippocampus (e.g., Houk and Wise, 1995; Schultz, 1998; Daw et al., 2005; Moustafa et al., 2009, 2010), and to simulate classical conditioning data and/or category learning data (e.g., Moustafa et al., 2009, 2010), or appetitive conditioning (for review, see Dayan and Balleine, 2002). Such models have also been successfully used to simulate shuttlebox avoidance (Johnson et al., 2002; Smith et al., 2004; Moutoussis et al., 2008; Maia, 2010) and can capture various features of empirical data including negatively accelerated learning curves, reduced latency to respond with extended training, and resistance to extinction when the shocks are no longer administered.

Here, we show that such a RL model incorporating actor and critic modules can also successfully capture many aspects of lever-press avoidance in SD rats, including the transition from escape to avoidance responding and the phenomenon of warm-up. The model thus provides one possible explanation of warm-up based purely on learning mechanisms, without requiring additional assumptions about motivational or emotional processes. We also show that WKY performance can be simulated by adjusting several parameters in the model, which have largely independent effects on aspects of avoidance. The model further predicts that SD will show warm-up, but WKY will show first-trial avoidance, under a range of inter-session intervals. As a partial test of this prediction, we tested SD and WKY rats in the lever-press paradigm with the inter-session interval reduced from the “standard” 48–24 h (daily sessions); results confirm the model predictions. The model therefore suggests that multiple, interacting mechanisms may underlie pathological avoidance in WKY rats, which in turn may provide insight into how such mechanisms could confer risk for anxiety disorders in humans.

## Modeling Methods

### Within-trial events

In a canonical version of the lever-press avoidance paradigm (e.g., Servatius et al., 2008), the warning signal *W* is a tone that comes on at the start of a trial and remains present for a 60-s warning period; a lever-press during this warning period is scored as an avoidance response and terminates the trial, triggering a 3-min safe period (ITI) signaled by a flashing light (*S*). Otherwise, once the 60-s warning period has elapsed, *W* remains on and scrambled 1 mA, 0.5 s footshocks (*U*) are delivered through the grid floor every 3 s for a maximum of 99 shocks. A lever-press during the shock period is scored as an escape response, terminating both *W* and *U*, and triggering the ITI. Twenty trials are typically delivered in a daily session, with sessions occurring on alternating days (48-hour inter-session interval); between sessions, the animal is removed to the home cage. Each session begins with a 60-s stimulus-free period in the testing chamber.

**Figure 1:**
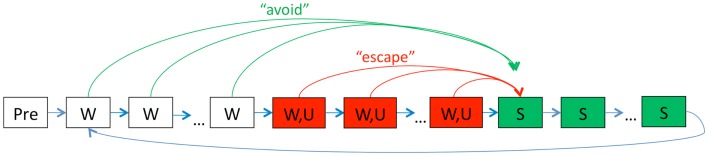
**Schematic of events during one acquisition session in the model**. Each training session begins with a short stimulus-free period (Pre) in the testing chamber context. Then, on each trial, the warning signal *W* is presented for several timesteps (“warning period,” white boxes), with each timestep representing about 10 s. Next, *W* and the shock *U* are presented together for several timesteps (“shock period,” red boxes); finally, both *W* and *U* are removed and the ITI signal *S* is presented for several timesteps (“ITI period,” green boxes), after which the next trial begins with another presentation of *W*. At each timestep, the actor module chooses and executes a response from a large set of possible actions; one of these is arbitrarily designated as lever-press. Lever-press during the warning period is scored as an avoidance response: in this case, *W* is terminated, *U* is omitted, and the trial proceeds directly to the ITI. Lever-press during the shock period is scored as an escape response: in this case, *W* and *U* are terminated and the trial proceeds directly to the ITI. Lever-press responses during the stimulus-free period at the start of the session (Pre) are scored as anticipatory responses. Events during extinction sessions are identical except that *U* is never presented.

To simulate this paradigm, each trial is divided into 54 timesteps that each represents approximately 10 s of simulated time. At each timestep, inputs signal the presence or absence of *W*, *S, U*, and the context (home cage or experimental chamber). The acquisition phase of the task consists of 12 sessions; Figure 1 shows a schematic representation of the events in one acquisition session. Under standard conditions, each acquisition session starts with six timesteps in the experimental context, followed by 20 trials. On each trial, *W* is presented for 6 timesteps (warning period) and persists through a further 30 timesteps where *U* is also presented (shock period), followed by 18 timesteps with *S* (ITI period).

At each timestep, the actor receives inputs and can choose a response from among a set of possible actions, with one action arbitrarily designated as lever-press. A lever-press response during the warning period, but before onset of shock, is scored as an avoidance response and terminates *W* and causes the trial to move directly to the ITI period; a lever-press response during the shock period is scored as an escape response and terminates *W* and *U*, and causes the trial to move directly to the ITI period.

Following the end of each session, an “overnight” period is simulated during which the home cage context input is present instead of the testing chamber context, no other inputs (*W*, *U, S*) are present, and the lever-press response is disabled. This overnight period is 18,000 timesteps in length, to simulate the relative ratio of home cage time to testing sessions in animals given testing sessions on alternating days.

The last acquisition session is followed by 12 extinction sessions that are the same as acquisition except that *U* is never presented.

### Actor module

**Figure 2:**
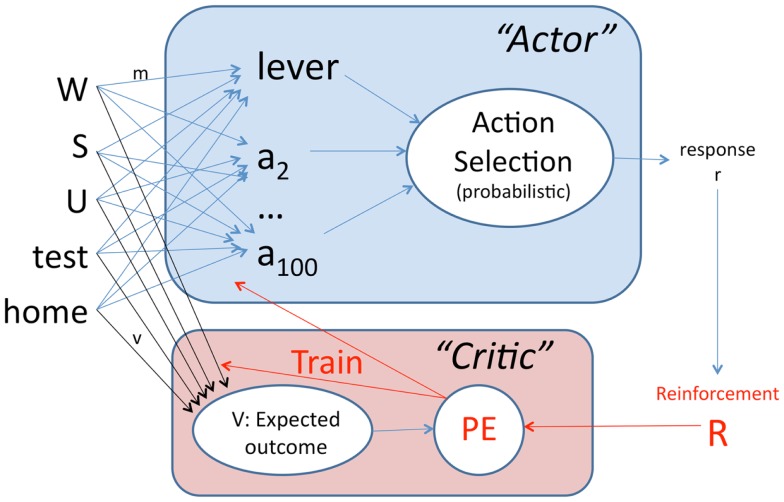
**Schematic of the actor–critic model**. Inputs consist of values indicating presence or absence of the warning signal *W*, the ITI signal *S*, the shock *U*, and the context (experimental testing chamber or home cage). The actor contains weighted connections from every input to each of several possible actions *a*_1_, …, *a*_100_, one of which is arbitrarily designated as lever-press. Based on the sum of weighted inputs, a probabilistic rule is used to select one action at each timestep. Reinforcement *R* is then provided to the critic module, which also contains weighted connections from each input, and calculates *V*, a prediction of future reward (or punishment). The prediction error *PE*, which is the difference between expected outcome *V* and actual outcome *R*, is then used to train the weights in both the actor and critic modules.

At every timestep *t*, the actor module chooses a response *r* from a set of *A* possible actions, of which one is arbitrarily designated to represent lever-press (Figure 2). To capture the fact that lever-press is only one of a large number of possible actions available to an animal (e.g., grooming, rearing), *A* = 100 in these simulations. The probability of selecting a particular response *r* at timestep *t* is defined as

$$\mathrm{Pr}\left(r\right)=\frac{f\left(\frac{M_{r}}{T}\right)}{\sum_{a}f\left(\frac{M_{a}}{T}\right)}$$

where *f*  (*x*) = *e^x^, a* = 1..*A* and *T* is an explore/exploit parameter (sometimes called the “inverse temperature”) which governs the tendency to repeat previously reinforced responses vs. explore the effect of new ones. At each timestep *t*, the values *M*_a_ are computed as

$$M_{a}=\sum_{\text{i}}m\left[a\right]\left[i\right]*I_{i}+p*c\left[a\right]\left[i\right].$$

Here, *I*_i_ is the current value of input *i*; *m*[*a*][*i*] is the strength of the connection from input *i* to action *a*, with all *m*[*a*][*i*] initialized to a small value (0.01) at the beginning of a simulation run. *P* is a perseveration factor governing the tendency to repeat a prior action (values of *P* < 0 confer a tendency for spontaneous alternation) and *c* is a working memory trace that records prior actions in response to the inputs: *c*[*r*][*i*] = 1 for the action *r* which was executed at time *t*; for all actions *a* ≠ *r, c*[*a*][*i*] ← *c*[*a*][*i*]*0.95. All *c*[*a*][*i*] are initialized to 0 at the start of a simulation run.

### Critic module

Based on the action *r* selected by the actor module at timestep *t*, external reinforcement *R* is provided. If shock is present at *t* + 1, then *R* is set to *R*_shock_, a large negative value (e.g., −4); otherwise *R* = 0 unless the action selected was lever-press, in which case *R* is set to *R*_press_, a small negative value (e.g., −0.2) representing the cost of lever-press in energy expenditure and missed opportunity to engage in other behaviors.

Based on *R*, the critic module computes prediction error *PE*, defined as

PE=R+0.9∗V−V′

where *V* is the predicted future value of *R*, calculated as

$$V=\sum_{\text{i}}v\left[i\right]*I_{i}$$

and where *V*′ is the value of *V* from the prior timestep. All *v*[*i*] are initialized to 0 at the start of a simulation run, and updated as

$$\Delta v\left[i\right]=\alpha *\mathrm{PE}*I_{i}$$

where α is a learning rate that governs rate of weight change in the critic. The values of *v*[*i*] are clipped at ±*R*_shock_, to prevent *v* from growing out of bounds.

The weights in the actor module *m*[*r*][*i*] for the chosen action *r* are also updated based on PE:

$$\Delta m\left[r\right]\left[i\right]=\varepsilon *\left(\mathrm{PE}-m\left[r\right]\left[i\right]\right)*I_{i}$$

where ε is the learning rate that governs rate of weight change in the actor. The values of *m* are restricted to be ≥0.

### Simulating behavior

For each trial, the dependent variables are the latency to first lever-press response on that trial (calculated in timesteps since the onset of *W*), and whether that first lever-press constitutes an avoidance response (occurring within the warning period), an escape response (occurring within the shock period), or neither (occurring during the ITI). If no lever-press responses are made during the trial, latency defaults to the maximum number of timesteps in the trial. In addition, anticipatory responses are defined as lever-press responses occurring during the stimulus-free period at the beginning of each session.

To simulate the behavior of SD rats, parameter space was explored for four free parameters: α (learning rate in the critic), ε (learning rate in the actor), *T* (explore/exploit), and *P* (perseveration). Parametric explorations are shown in the Supplementary Material; in brief, manipulations of *T* tended to affect rate of avoidance acquisition, without much effect on extinction or warm-up; manipulations of α tended to affect rate of extinction, without much effect on acquisition or warm-up; and manipulations of *P* tended to affect warm-up without much effect on either acquisition or extinction. Manipulations of ε also tended to affect acquisition rate, but these effects were more dramatic than the effects of manipulating *T*, and realistic learning curves were only obtained within a fairly small range of values. Simulations that best simulated key features of SD behavior were obtained when α = 0.05, ε = 0.005, *T* = 1.0, and *P* = 0.25, and these values were subsequently “fixed” for the SD simulations reported below.

Next, the model was adjusted to simulate behaviorally inhibited WKY rats. While WKY rats have a number of phenotypic differences compared to control strains, there are three in particular that appear to relate in a fairly straightforward way to RL model parameters. First, because WKY rats are behaviorally inhibited, and behavioral inhibition implies a tendency to repeat previously reinforced (familiar) responses rather than explore new ones, we reduced the value of *T*. Second, given data suggesting that WKY rats have reduced mesolimbic dopamine function (Jiao et al., 2003), a system which has been implicated in generating the prediction error signal in RL (Hollerman and Schultz, 1998; Schultz and Dickinson, 2000), we reduced the learning rate α at which the critic updates weights based on prediction error. Third, given data suggesting that WKY rats have reduced dopamine function in prefrontal cortex (De La Garza and Mahoney, 2004), a brain area implicated in working memory, such as would maintain a trace of recent responses (Goldman-Rakic, 1992; Bussey et al., 2001), we reduced the perseveration parameter *P*. As described below, simulations with these three parameter values (i.e., α = 0.005, *T* = 0.25, and *P* = 0), produced behavior that simulated key features of the WKY rat.

All modeling results reported are averaged over 10 simulation runs.

## Modeling Results

### Basic features of avoidance acquisition and extinction in SD and WKY

**Figure 3:**
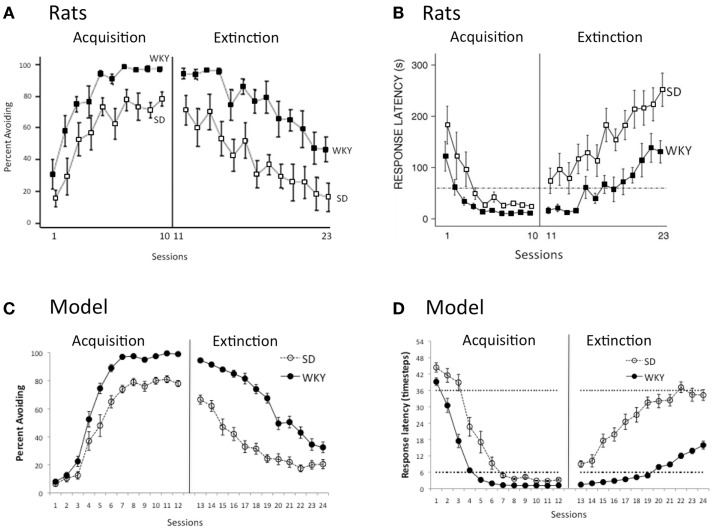
**Acquisition and extinction of avoidance**. **(A)** Male WKY rats acquire avoidance, expressed as percent of trials with an avoidance response, faster (sessions 1–10) and to a higher asymptotic level, and extinguish slower (sessions 11–23), compared to male SD rats. Adapted from Figure 5 of Jiao et al. (2011a). **(B)** The same strain difference is reflected in latency to respond: male WKY rats respond faster than male SD rats during acquisition, and continue to give short-latency responses during the first few extinction sessions. Here, latency is defined as average time from onset of warning signal to first avoidance response; responses occurring within first 60 s after warning signal onset (below dotted line) are avoidance responses. Adapted from Figure 1 of Servatius et al. (2008). **(C)** As in the rat data, the WKY model acquires faster (sessions 1–12) and extinguishes slower (sessions 13–24) than the SD model. **(D)** Similarly, the WKY model gives faster latency responses than the SD model, and continues to give short-latency avoidance responses for the first several sessions of extinction. Avoidance responses occur within the first six timesteps after warning signal onset (below dotted line). Here and in subsequent figures, simulation results are shown averaged over 10 simulation runs; error bars show SEM computed across runs.

Figure 3A shows typical acquisition and extinction curves obtained in male SD and WKY rats, expressed as percent of trials with an avoidance response, with WKY rats acquiring faster (sessions 1–10) and to a higher asymptotic level, compared to SD rats (Jiao et al., 2011a); WKY rats also extinguish slower when shock no longer occurs (sessions 11–23). Another way to assess learning is by considering latency from onset of the warning signal to first lever-press response; responses occurring before shock onset (during the warning period) are avoidance responses, and those occurring during the subsequent shock period are escape responses. As shown in Figure 3B, during the first few acquisition sessions, both SD and WKY rats rapidly decrease average latency, so that on most trials, responses occur within the warning period; during extinction, latency rapidly increases in SD rats while WKY rats continue to give responses during the warning period for several sessions, even though the shock no longer occurs (Servatius et al., 2008). Figure 3C shows acquisition and extinction curves obtained in the SD and WKY models, with fast acquisition and slow extinction in the WKY model. Similarly, the SD model shows decreasing response latency across the 12 acquisition sessions, so that by the end of acquisition, most responses are avoidance responses that occur within the warning period (here, within timesteps 0–6); during extinction, latencies quickly increase (Figure 3D). However, in the WKY model, response latencies remain within the warning period for several extinction sessions, similar to the rat data shown in Figure 3B.

**Figure 4:**
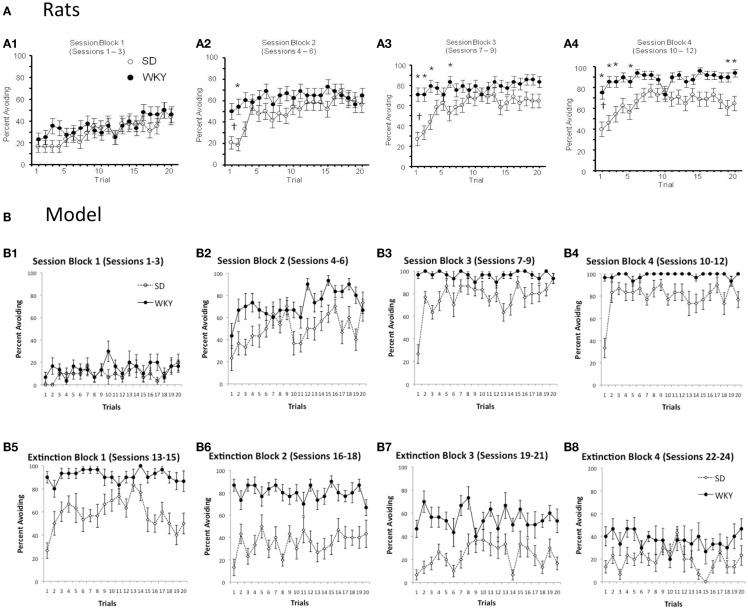
**Warm-up – transiently decreased avoidance responding at the start of a session, compared to the end of the previous session or later in the same session – plotted as trial-by-trial responding, averaged over blocks of 3 sessions**. **(A)** Warm-up is exhibited by SD but not WKY rats. Over the first three sessions of acquisition **(A1)**, both SD and WKY rats show increased avoidance responses across trials within a session. Over later session blocks **(A2–4)**, SD rats show warm-up, but WKY rats generally start each session at about the same performance level as at the end of the prior session. Asterisks indicate significantly greater responding in WKY than SD; crosses indicate significantly less responding by SD on the first two trials of a block than on the last two trials of the preceding block. Adapted from Figure 1 of Perrotti et al. (2013). **(B)** Similarly, warm-up is shown during acquisition by the SD but not WKY model **(B1–4)**. During early extinction sessions **(B5,6)**, the SD model continues to show warm-up: lower response rates at the beginning of a session than at the end of the prior session or later in the same session, even though no shocks are provided during these sessions.

As mentioned above, warm-up is exhibited in the SD but not WKY rats. Figure 4A shows typical within-session avoidance responding patterns, plotted as trial-by-trial responding averaged across several blocks of training sessions (Perrotti et al., 2013). As illustrated in the figure, avoidance responding typically increases across trials within a session, but particularly in later sessions, SD rats generally make fewer avoidance responses on the first few trials of a session, compared to their performance at the end of the previous session or later in the same session. Figure 4B shows similar within-session data from the SD and WKY model. During the first three sessions of acquisition, the SD model does not show much avoidance responding (Figure 4B1); however, as the avoidance response is acquired in sessions 4–6 and beyond, the SD simulations reliably show warm-up (Figures 4B2–4). WKY simulations do not show warm-up during these acquisition sessions. During early extinction (Figures 4B5,6), the SD model continues to show warm-up, meaning that avoidance responses increase over the first few trials of an extinction session, even though no reinforcer is delivered; this pattern of paradoxical increases in responding across the first few trials of early extinction session has also been observed in SD rats (Beck et al., 2011).

### Effects of manipulating shock intensity

**Figure 5:**
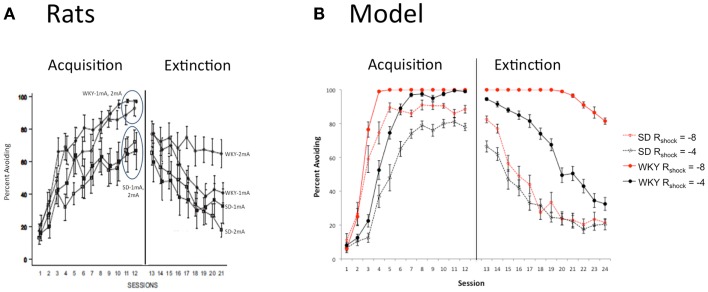
**Effects of punisher intensity**. **(A)** The facilitated acquisition in WKY rats is independent of shock intensity (1 vs. 2 mA), although male WKY rats trained with the 2 mA shock extinguish more slowly than counterparts trained with 1 mA shock, or SD rats at either intensity. Adapted from Figure 1 of Jiao et al. (2011b). **(B)** In the model, shock intensity is determined by the value of *R*_press_. As in the animal data, increasing the shock intensity (from *R*_press_ = -4 vs. -8) strongly attenuates extinction in the WKY model, with relatively little effect on extinction in the SD model.

One possible reason for faster learning in the WKY strain could be increased sensitivity to shock, since stronger punishers should tend to produce faster associative learning. However, increasing the shock amplitude, e.g., from 1 to 2 mA, does not significantly alter acquisition speed in either WKY or SD rats, with SD rats continuing to learn more slowly than WKY rats at either amplitude (Figure 5A; Jiao et al., 2011b), although extinction in the WKY rats is worse after training with the higher amplitude shock. Figure 5B shows a similar pattern in the model: when the shock amplitude (value of *R*_shock_ in the model) is doubled, WKY simulations still learn faster than SD simulations; however, extinction in the WKY model is severely attenuated following training with the greater shock amplitude. The modeling results suggest that differences in shock sensitivity do not have to be assumed to explain strain differences in learning and extinction.

### Effects of manipulating warning signal

**Figure 6:**
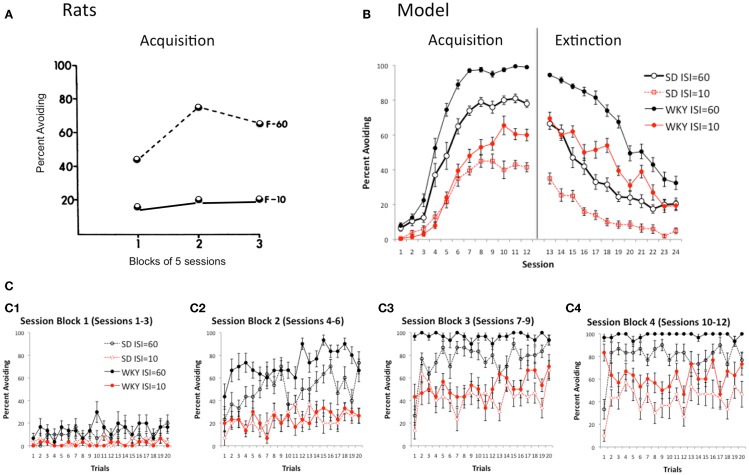
**Effects of ISI**. **(A)** On a lever-press procedure, female SD rats could learn with a 60 s ISI (F-60) but not a 10 s ISI (F-10). Adapted from Figure 2 of Berger and Brush (1975). **(B)** Acquisition is similarly reduced in both the SD and WKY model when the ISI is reduced from 60 to 10 s; however, even at the shorter ISI, the WKY model still learns faster and extinguishes more slowly than SD model. **(C)** Presence of warm-up (SD) and lack of warm-up (WKY) is not affected by ISI in the model.

In outbred rat strains such as SD, learning of lever-press avoidance is affected when the length of the warning signal (interstimulus interval or ISI) is varied (Cole and Fantino, 1966; Berger and Brush, 1975; Berger and Starzec, 1988). For example, on a lever-press avoidance task similar to the paradigm described above, SD rats trained with a fixed-interval 60-s warning signal (F-60) acquired the avoidance response, but those trained with a 10-s warning signal (F-10) exhibited low levels of avoidance responding, although escape responding was robust (Figure 6A; Berger and Brush, 1975). Reduced avoidance responding under the 10-s ISI is sometimes attributed to motivational factors, such as a fear response to the warning signal which causes freezing that must be overcome before lever-pressing can be initiated; such explanations assume that a 60-s ISI is enough to allow this fear response to dissipate but a 10-s ISI is not. However, such explanations need not necessarily be invoked to explain reduced avoidance acquisition under a shorter ISI. Specifically, when ISI in the SD model is reduced from 60 s of simulated time to 10 s, avoidance acquisition is greatly reduced, although not abolished (Figure 6B). This is simply due to the probabilistic nature of response selection in the actor module of the model; with a longer ISI there is greater probability that lever-press will be selected at least once during the warning period, compared to a shorter ISI which provides fewer timepoints at which to select actions. On the other hand, WKY rats can acquire robust avoidance responses even under the shorter ISI (Berger and Starzec, 1988); Figure 6B shows that the WKY model is less impaired under the 10 s ISI than is the SD model, although performance is not as good as under the longer ISI for either model.

Manipulating the ISI provides a way to explore another possible explanation for the absence of warm-up shown in WKY rats and the WKY model, which is that warm-up occurs only while the avoidance response is still being acquired; thus, SD rats (and model) which learn slowly continue to show warm-up behavior throughout the acquisition sessions, but WKY rats (and model) which quickly reach a higher level of performance do not show warm-up behavior. However, Figure 6C shows that, while the SD model shows warm-up under both the 10 and 60-s ISI conditions, there continues to be an absence of warm-up in the WKY model, even under the 10-s condition, where a relatively low performance criterion is reached even in the final session block of acquisition training (Figure 6C4).

The model therefore makes the novel prediction that the presence of warm-up in SD, and the absence of warm-up in WKY, should be independent of whether high or low performance levels are reached.

### Manipulations of inter-session interval

**Figure 7:**
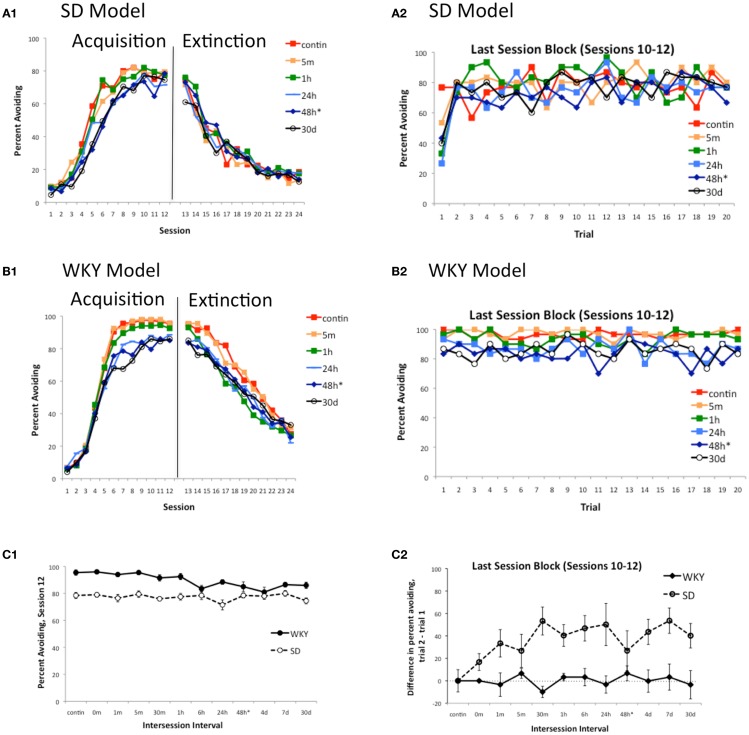
**Warm-up as a function of inter-session interval in the model**. Acquisition and extinction were simulated under a range of inter-session intervals in the SD and WKY models. There is little effect of inter-session interval on acquisition or extinction in the SD model **(A1)**, while for the WKY model **(B1)**, asymptotic responding (in session 12) is lower when inter-session interval is more than about 1 h of simulated time. For illustration, learning curves obtained under a few intervals are shown in **(A1)** and **(B1)**; **(C1)** shows the eventual asymptote (percent avoiding, session 12) for all intervals tested, in both the SD and WKY models. Inter-session interval does affect warm-up in the SD but not WKY model; again, for illustration **(A2)** and **(B2)** show data obtained under a few intervals while **(C2)** plots the average change in response rate from trial 1 to trial 2 of the last session block, at each interval tested (up to 30 days of simulated time). In the SD model **(A2)**, warm-up is absent if sessions are continuous (no inter-session interval), but some warm-up is observed when even a short inter-session interval is interposed, and warm-up is robust when the inter-session interval is 30 min or longer. Warm-up is not evident in the WKY model at any inter-session interval tested. *contin* = continuous sessions, *m* = min, *h* = hours, *d* = days; * = “standard” inter-session interval. Note that, for all conditions except *contin*, each session started with a 1 min pre-stimulus interval, in addition to the explicit inter-session interval.

Another feature of warm-up observed in early studies with outbred rats is that it appears even when the inter-session interval is fairly short, e.g., 30 min (Hineline, 1978) or 1 h (Kamin, 1963), and occurs whether or not the animal is removed to the home cage between sessions, or is housed round-the-clock in the conditioning chambers to eliminate possible contextual effects (Hineline, 1978). The SD model is able to capture these effects as well. As the length of the inter-session is varied from 0 min to the “standard” 48 h, and even up to the equivalent of 30 days of simulated time (259,200 timesteps) between testing sessions, there is little effect on acquisition or extinction rate in the SD model (Figure 7A1; for clarity, only a few representative curves are shown); Figure 7C1 plots the eventual asymptote (avoidance rates in training session 12) for all values of inter-session interval explored in the model, and shows that all simulations reached approximately the same asymptote. However, inter-session interval does affect warm-up in the SD model, evident as a sharp decrease in response on the first trial of a session (Figure 7A2; again, for clarity, only a few representative curves are shown); Figure 7C2 shows data from all inter-session intervals explored, plotted as a difference score representing the average difference in responding on trial 2 vs. trial 1 of sessions 10–12. There is no warm-up in the SD model when sessions are continuous, but warm-up emerges with inter-session intervals as short as a few minutes of simulated time, and reaches what appears to be a maximum with intervals of 30 min or longer. The same general pattern of results is obtained in the SD model when “round-the-clock” housing is simulated; i.e., when contextual inputs remain the same throughout the experiment rather than switching to the home cage context during the inter-session interval (simulations not shown). Therefore, the model can also capture this feature of warm-up in the SD rat.

On the other hand, the model predicts that changes in inter-session interval will affect acquisition and eventual asymptote in the WKY, without producing warm-up. Figure 7B1 shows that shorter inter-session intervals (e.g., ≤1 h of simulated time) produce faster learning to a higher asymptote, and slower extinction, than longer inter-session intervals (e.g., ≥6 h). However, in no case do WKY simulations exhibit warm-up (Figure 7B2).

The simulations with varying inter-session interval have implications for empirical studies. In particular, lever-press avoidance in rats is typically run with sessions on alternating days (i.e., 48-hour inter-session interval); this is primarily due to a tacit assumption that more frequent sessions (e.g., 24-hour inter-session interval) might be too stressful or otherwise impair learning. However, the simulations in Figure 7 suggest that, over a wide range of inter-session interval, there is little effect on acquisition, extinction, or warm-up in either the SD or WKY model, at least for inter-session intervals longer than about 30 m. In particular, if the model can adequately account for the major processes underlying avoidance learning in SD and WKY rats, then data obtained under daily training should show the same basic features of faster acquisition in WKY, with SD but not WKY showing warm-up. This prediction was tested with an empirical study, as described next.

## Empirical Methods

As a test of the model prediction that strain differences in acquisition and warm-up observed under the “standard” inter-session interval of 48 h appear also with a 24-hour inter-session interval, an empirical study was conducted with SD and WKY rats given daily training sessions of lever-press avoidance. Materials and procedures generally followed those of prior studies described above (Servatius et al., 2008; Beck et al., 2010) except for inter-session interval which was reduced to 24 h, as described below. The study methods were approved by the IACUC at VA New Jersey Health Care System and confirmed to Federal standards set in the NIH Guide for the Care and Use of Laboratory Animals.

### Animals

Eight male WKY rats (10 weeks old) and 8 male SD rats (10 weeks old) were obtained from Harlan Labs Inc. (Indianapolis, IN, USA). Rats were individually housed in cages on a 12:12 light cycle (lights on at 0700). All rats had at least 2 weeks to acclimate to their living conditions prior to the start of training and had free access to water and food in their home cages. The Institutional Animal Care and Use Committee approved all procedures in accordance with AAALAC standards.

### Apparatus

Training was conducted in 30 cm × 25 cm × 30 cm operant avoidance chambers. The chambers were sound attenuated and had clear Plexiglas front doors. One wall was fitted with a lever (10.5 cm above the grid floor), a speaker (26 cm above the floor), and a light cue (20.5 cm above the floor) that designated the ITI, and blinked at a rate of 0.5 Hz when illuminated. On the opposing wall, a house light (26 cm above the floor) was continually lit for illumination. A scrambled 1.0 mA electric footshock was delivered via a shocker (Coulbourn Instruments, Langhorn, PA, USA).

### Avoidance conditioning

Twelve acquisition sessions occurred during the light cycle over twelve consecutive days. Each session began with a 1 min stimulus-free period, followed by 20 escape-avoidance trials. A trial began with a 75 dB, 1000 Hz tone (warning signal) that preceded the first shock by 1 min. Lever-press responses during this tone-alone warning period terminated the tone and were scored as avoidance responses. If no avoidance response was made, the tone remained on and a series of 1.0 mA footshocks (0.5 s in duration every 3 s) were delivered through the grid floor; lever-press responses during this period caused termination of both tone and shock and were scored as escape responses. In the absence of an escape response, shocks terminated after 300 s. Each trial was followed by a 3 min ITI, during which the blinking light cue (ITI signal) was presented. Typically, any rats that fail to produce at least five lever-press responses by the end of Session 5 are excluded; in the current experiment, no animals met this criterion and none were excluded.

### Data analysis

Graphic State (Coulbourn Instruments, Langhorn, PA, USA) was used to control the testing apparatus and to record avoidance responses and response latency on each trial. Custom algorithms in S-Plus were used to detect all actions on the lever during the entire session. Avoidance responses were ascertained from these data, and they were analyzed using mixed-design ANOVA with between-subjects factor of strain and between-subjects factor of trial and/or session.

## Empirical Results

**Figure 8:**
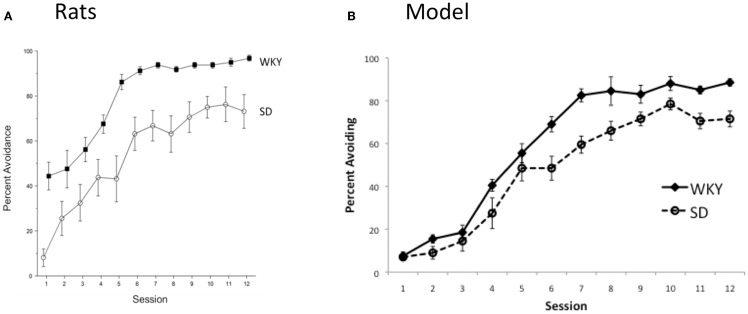
**Avoidance acquisition under “daily” training (i.e., 24 h inter-session intervals)**. **(A)** Empirical results show that the strain difference is preserved, with WKY rats learning the avoidance response faster, and to a higher asymptote, than SD rats. **(B)** For comparison, model predictions for this condition are replotted here from Figure 7. Consistent with the empirical data, the WKY model shows facilitated acquisition relative to the SD model.

Given daily testing sessions (24-h inter-session interval), there were main effects of Strain, *F*(1, 14) = 33.5, *p* < 0.0001 and Session, *F*(11,154) = 32.9, *p* < 0.0001, indicating acquisition of the avoidance response occurred in both strains, but the strains differed in their overall performance (Figure 8A). WKY rats acquired the avoidance behavior quicker and to a higher asymptotic level than SD. Thus, as in the model (Figure 8B), decreasing the inter-session interval from 48 to 24 h preserved the faster acquisition normally observed in WKY rats.

**Figure 9:**
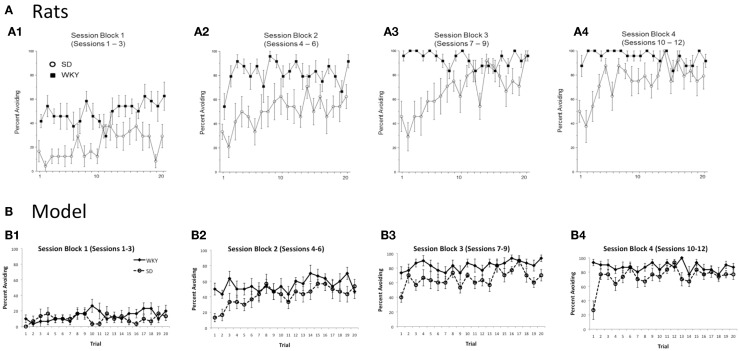
**Within-session responding under “daily” training**. **(A)** Empirical data show that warm-up is preserved in SD rats under daily training, while WKY rats show response rate on the first trials of a session comparable to their levels at the end of the prior session. **(B)** For comparison, model predictions for this condition are replotted here from Figure 7; consistent with the empirical data, the SD model but not WKY model shows warm-up.

Next, to examine effects of the shorter inter-session interval on warm-up, avoidance responses were analyzed within a session, averaged across three sessions for each of four session blocks. There were main effects of strain, *F*(1, 14) = 33.5, *p* < 0.0001, Session block, *F*(3,42) = 56.8, *p* < 0.0001, and trial, *F* (19, 266) = 5.1, *p* < 0.0001, as well as an interaction between strain and trial, *F*(19,266) = 5.1, *p* < 0.0001); specifically, as shown in Figure 9A, WKY rats tended to outperform SD rats, particularly on the early trials of a session; by the later trials of a session block (particularly later session blocks, Figures 9A3,4), SD rats approximated the performance levels of WKY rats. As evidenced by the average of the first two trials of the last session block vs. the last two trials of the previous session block, WKY rats show absolutely no evidence of warm-up, whereas the SD rats clearly exhibit warm-up. Thus, the empirical data support the model predictions (Figure 9B) that warm-up is preserved in SD rats, but absent in WKY rats, even under the shorter inter-session interval.

## Discussion

The current work demonstrates that a RL model can capture many aspects of avoidance acquisition and extinction of lever-press responding in outbred SD rats, including the phenomenon of warm-up, which correctly appears in the SD model even when the inter-session interval is fairly short (≥30 min of simulated time) and even if inter-session intervals occur in the training environment, with no context shift (removal to home cage) between sessions. As in the empirical data, warm-up in the SD model does not require explanations invoking emotional effects, contextual shift effects, or simple forgetting; rather, warm-up in the SD model reflects a tendency to perseverate or repeat behaviors that have occurred during the inter-session interval at the expense of avoidance responding, similar to the interpretation proposed by Spear et al. (1973). Thus, when the parameter *P*, which governs perseveration, is reduced to 0, warm-up is abolished without much effect on other aspects of behavior in the model, such as rate of acquisition or extinction (see Figures S1C,D in Supplementary Material).

The model also provides an explanation of the finding that SD rats show reduced avoidance acquisition under short ISI. This poor learning is sometimes attributed to motivational factors such as a fear response to the warning signal that causes freezing which must be overcome before an operant avoidance response can be initiated; under this theory, the shorter ISI simply does not leave enough time for this emotional response to dissipate before shock onset. However, the model provides a simpler interpretation: a shorter warning signal is simply shorter, making it less likely that a probabilistic response selection process will choose a lever-press response at least once within that time period, compared to the probability under a longer warning signal.

The model can also address data from behaviorally inhibited WKY rats, which typically show faster acquisition, slower extinction, and lack of warm-up. WKY-like behavior is produced when the model is altered by reducing the default values of three model parameters: reducing the explore/exploit parameter *T*, which causes a decrease in behavioral exploration similar to behavioral inhibition, and increases acquisition rates; reducing the learning rate α, which impairs extinction; and reducing the perseveration parameter *P*, which reduces warm-up. The model also correctly captures the effect of increasing the intensity of the punisher, which causes little facilitation of acquisition in either rat strain but greatly retards extinction in the WKY rats.

The ability of the model to simulate these strain differences suggests that differences in behavior between SD and WKY rats may be best understood as resulting from distinct associative learning mechanisms, each of which may be amenable to independent study. If the mechanisms underlying pathological avoidance in WKY rats are similar to those underlying avoidance vulnerability in humans, then avoidance vulnerability may similarly reflect a confluence of several mechanisms which, together, produce the endophenotype.

The RL model also makes several novel predictions. First, it predicts that the impaired extinction observed in WKY rats is not simply an artifact of their higher response asymptote during acquisition, compared to SD rats. Instead, even under a short ISI where a fairly low response asymptote is reached during acquisition, the WKY model continues to show impaired extinction compared to the SD model trained under the same conditions (Figure 6).

Second, the model predicts that the accelerated avoidance in WKY rats is not simply a reflection of the absence of warm-up. As shown by parametric manipulations (Figures S1C,D in Supplementary Material), altering the perseveration parameter *P* at least within a range from neutral (*P* = 0) to mildly positive (*P* ≤ 0.25) values affects warm-up but has little effect on rates of either acquisition or extinction of avoidance responding. Even under conditions where the WKY model shows degraded learning, such as the short ISI training simulated in Figure 6, the SD model nevertheless still shows warm-up, and the WKY model does not.

Third, while continuous sessions abolish warm-up, for inter-session intervals ranging from 30 min to 30 days of simulated time, warm-up is robust in the SD model, but never appears in the WKY model. This prediction was partially confirmed by our empirical data, which show that when the inter-session interval is halved, from the “standard” 48 to 24 h (daily sessions), WKY rats still acquire the avoidance response faster than SD rats, while SD but not WKY still show warm-up. While the daily testing sessions may arguably be more stressful for the animal, in neither the empirical study nor the model simulations did this change affect associative learning.

Limitations of the current work include the fact that the RL model is a fairly abstract model; although parameters can be manipulated which bear some resemblance to known features of SD vs. WKY rats, the RL model cannot provide a complete account of the underlying biology that gives rise to strain differences in avoidance behavior. In addition, while the current study focused on comparing SD and WKY, there are other strain differences that could be modeled. For example, the outbred C57BL mouse strain appears to acquire a lever-press avoidance response about as well as outbred SD rats, but an inbred strain, the FVB/NJ mouse, learns to escape but not avoid (Brennan, 2004). The RL model could be used to examine possible mechanisms underlying this behavioral phenotype, which may be relevant to understanding comparable phenotypes in human anxiety and depression.

Further, although strain is indeed an important determinant of variability in learning and behavior, there are other important individual differences that affect acquisition and maintenance of avoidance too; among these are sex differences (Beck et al., 2010, 2011), which the current model does not address, although some aspects of sex differences might be in principle amenable to future study to determine which parametric differences best capture behavioral differences observed between male and female rats. In particular, while female rats generally outperform male rats of the same strain on lever-press avoidance acquisition, male and female rats are differentially affected by the presence of the safety signal during the ITI (Beck et al., 2011), and computational modeling might help elucidate some of the mechanisms underlying this difference.

Finally, although the RL model provides simple explanations for many features of avoidance that do not require invoking motivation or emotion as constructs, nevertheless SD and WKY rats clearly differ in emotional responding; in fact, one of the defining characteristics of behavioral inhibition in WKY rats is exaggerated freezing after initial placement in the center of a brightly lit open field or when faced with an electrified probe [for review, see Jiao et al. (2011a)]. Such freezing would obviously be expected to facilitate passive avoidance in WKY rats, although it would actually be expected to impair – not facilitate – active avoidance compared to SD rats. Although freezing to the warning signal has not to our knowledge been explicitly assessed in WKY rats during lever-press avoidance, there are no differences between WKY and SD rats in freezing to a tone stimulus that has been paired with an electric shock in a classical conditioning paradigm (LeDoux et al., 1983). In addition, increasing the shock intensity, which should presumably increase emotional responding, does not greatly affect acquisition in either strain (Figure 5A; Jiao et al., 2011b). For these reasons, freezing alone does not appear to adequately explain the strain differences in warm-up. However, freezing is an important species-specific response to threatening stimuli, and may play an important role in strain differences in active avoidance; in fact, given the higher freezing in WKY rats placed in the open field, it is theoretically possible that manipulations which reduce freezing would actually magnify strain differences observed in avoidance acquisition and extinction. On the other hand, avoidance learning is known to be facilitated following exposure to stressors (Brennan et al., 2005, 2006). The existing RL model does not consider how learning might be modulated by emotional and/or neurochemical states brought on by prior experiences, and thus it cannot directly address these concepts. However, the model simulations and empirical study both suggest that reducing inter-session interval, which might arguably cause an increase in stress – by increasing absolute shock frequency and/or allowing less time for arousal to dissipate between sessions – is not of itself sufficient to affect strain differences in avoidance acquisition and warm-up.

Future modeling work could address some of these ideas. Despite these limitations, the current work shows that a fairly simple RL model can simulate key features of lever-press avoidance, and parametric manipulations can capture a range of observed phenomena in acquisition, extinction, and warm-up, without needing to invoke additional motivational or emotional mechanisms. The model may thus provide a framework for further exploration of these mechanisms and their role in pathological avoidance, and in future could be used to explore the space of possible potential pathways (e.g., behavioral interventions) to remediate pathological avoidance. Such exploration can be done cheaply and quickly in a computational model, and paradigms identified as of interest could then be targeted for future study in rat models and also in humans. This in turn might help in the development of more sophisticated behavioral therapies to promote extinction of pathological avoidance or even prevent the initial development of pathological avoidance in anxiety-vulnerable individuals.

## Author Contributions

Catherine E. Myers, Kevin D. Beck, and Richard J. Servatius contributed to the design of the modeling work; Ian M. Smith and Kevin D. Beck contributed to the design and implementation of the empirical study. Catherine E. Myers conducted the computational modeling and model analysis; Ian M. Smith and Kevin D. Beck conducted the empirical data collection and analysis. All authors contributed to drafting and revising the manuscript, approved the final version, and agree to be accountable for all aspects of the work.

## Conflict of Interest Statement

The authors declare that the research was conducted in the absence of any commercial or financial relationships that could be construed as a potential conflict of interest.

## Supplementary Material

The Supplementary Material for this article can be found online at http://www.frontiersin.org/Journal/10.3389/fnbeh.2014.00283/abstract
