# Supplementary material for: Absence of “Warm-Up” during Active Avoidance Learning in a Rat Model of Anxiety Vulnerability: Insights from Computational Modeling
Source: Front Behav Neurosci. 2014 Aug 18;8:283. doi: 10.3389/fnbeh.2014.00283 (PMC4135546; doi:10.3389/fnbeh.2014.00283)
Supplement: Supplementary file 1 [file Presentation1.PDF]

# Supplementary Material

# Absence of "warm-up" during active avoidance learning in a rat model of anxiety vulnerability: Insights from computational modeling


Myers, Catherine E.[1,2*], Smith, Ian M.[1], Servatius, Richard J.[1,2], & Beck, Kevin D.[1,2]

[1] Department of Veterans Affairs, VA New Jersey Health Care System, East Orange, NJ, USA

[2] Stress & Motivated Behavior Institute, Department of Neurology & Neurosciences, New Jersey Medical School, Rutgers, The State University of New Jersey, Newark, NJ, USA

* **Corresponding Author**:
Catherine E. Myers
Research Scientist, NeuroBehavioral Research Lab
VA New Jersey Health Care System
385 Tremont Avenue, Mailstop 127A
East Orange, NJ 07042
Phone: 973-676-1000 Ext. (1) 1810
Email: Catherine.Myers2@va.gov

## Supplementary Material

In determining model parameters (*T*, *P*, α, ε, and $R_{shock}$) for the SD simulations, parameter space was explored, looking at configurations of possible parameters. However, in general, it appeared that individual parameters had fairly separable effects on model behavior; for example, as described below, the explore/exploit parameter *T* primarily affected acquisition rates, with little effect on extinction or warm-up, regardless of the other parameter values, while the perseveration parameter *P* primarily affected warm-up, with little effect on acquisition or extinction rates. As a result, for simplicity, the parameters are discussed individually below, and results are shown for varied levels of each parameter, holding the other parameters constant at the final levels used in the SD model ($T$=1.0, $P$=0.25, α=0.05, ε=0.005, $R_{shock}$ = -4).

In Figures S1-S3, these "standard" parameter values used in the SD model are indicated with an asterisk and plotted in green. Warm-up curves show responding on the third session block of acquisition (sessions 7-9), when most simulations reached asymptotic performance; in all cases, if warm-up was present (or absent) on this session block, the same was generally true on the final session block of acquisition (sessions 10-12, not shown).

### Explore/exploit parameter, *T*

Figures S1A and S1B show the effect on acquisition, extinction and warm-up of changing *T* through a range of values from a fairly low value ($T$=0.5), which favors exploration, to a neutral value ($T$=1), to a fairly high value ($T$=2), which favors exploiting or repeating previously-reinforced responses. Optimal acquisition is obtained with moderate values of *T*, while more extreme (high or low) values produce poorer acquisition; there is little effect on extinction or warm-up beyond that resulting from different acquisition rates. As a result, for the SD model, *T* was fixed at 1, representing a balance between tendencies to explore vs. exploit.

Figure S1

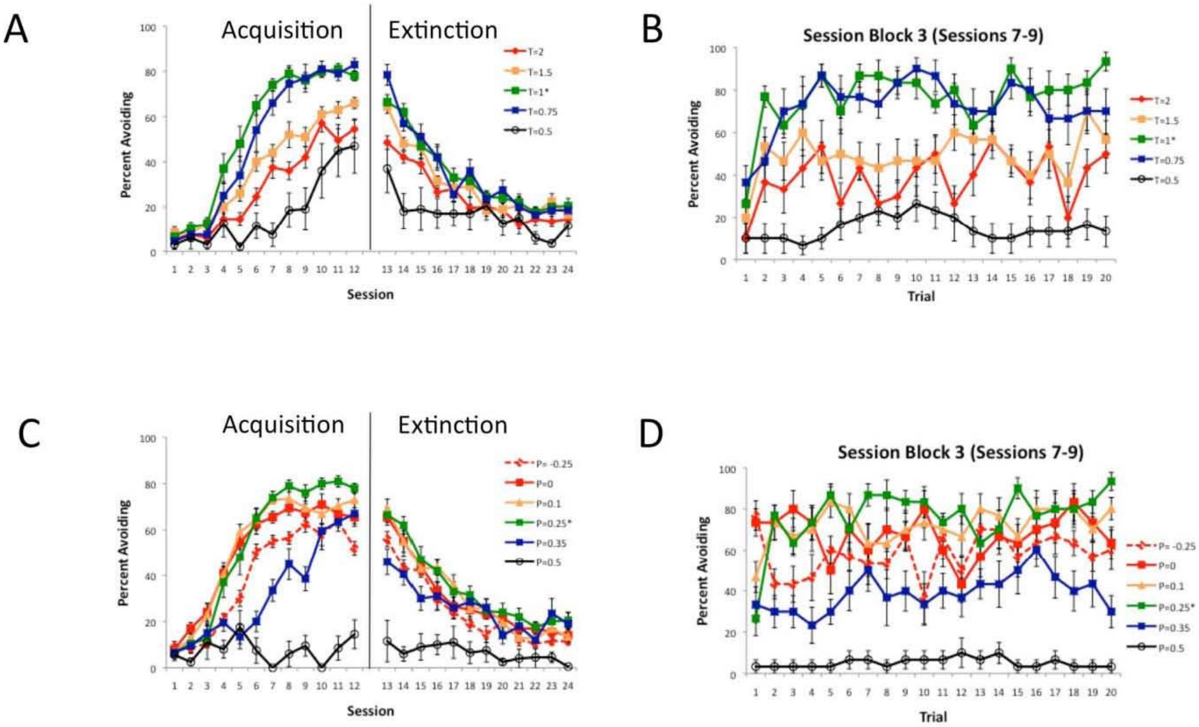


Figure S1. Parametric manipulations of the explore/exploit parameter *T* and the perseveration parameter *P* in the model. (A) There is good acquisition for values of *T* near 1, with poorer acquisition for very high or very low levels of T, and little effect on extinction; (B) for values of *T* that produce learning (e.g. *T*>0.5), there is little effect of *T* on warm-up, illustrated here in the third session block (sessions 7-9). (C) Mildly positive values of the perseveration parameter *P* produce good acquisition and also produce warm-up (D), although larger values (e.g. *P*=0.5) cause so much perseveration that learning degrades. Neutral values (*P*=0) also produce good acquisition and extinction, but no warm-up, while negative values (e.g. *P*= -0.25) produce a paradoxical tendency to respond more on the first trial than on the later trials of a session.

### Perseveration parameter, *P*

Figure S1C shows that neutral or small positive values of *P* ($0 \leq P \leq .25$) produce fairly rapid acquisition, although larger values of *P* degrade learning and negative values of *P* produce spontaneous alternation which strongly degrades acquisition. In all cases, there is little effect on extinction. However, S1D shows a strong effect of *P* on warm-up: specifically, warm-up appears for $0 < P \leq .25$ and is absent when *P*=0; for *P*<0, there is "inverse warm-up" – a tendency to respond more on the first trial of a session than on later trials. To capture the warm-up phenomenon observed in SD rats, the SD model has *P*=0.25, representing a moderate tendency to perseverate.

## Learning rates α and ε

The parameter α governs the rate at which the critic updates its expectations of future reinforcement. For a range of values of α, there is not much effect on acquisition (Figure S2A) or warm-up (Figure S2B); however, there is a strong effect on extinction: specifically, extinction is fastest when α is high, while very low values (e.g. α=0.001) essentially prevent extinction, as the critic does not update its weights to reflect omission of expected reinforcement during the extinction trials. To capture the relatively efficient extinction in SD rats, α is set to an intermediate value of 0.05 in the SD model.

Figure S2

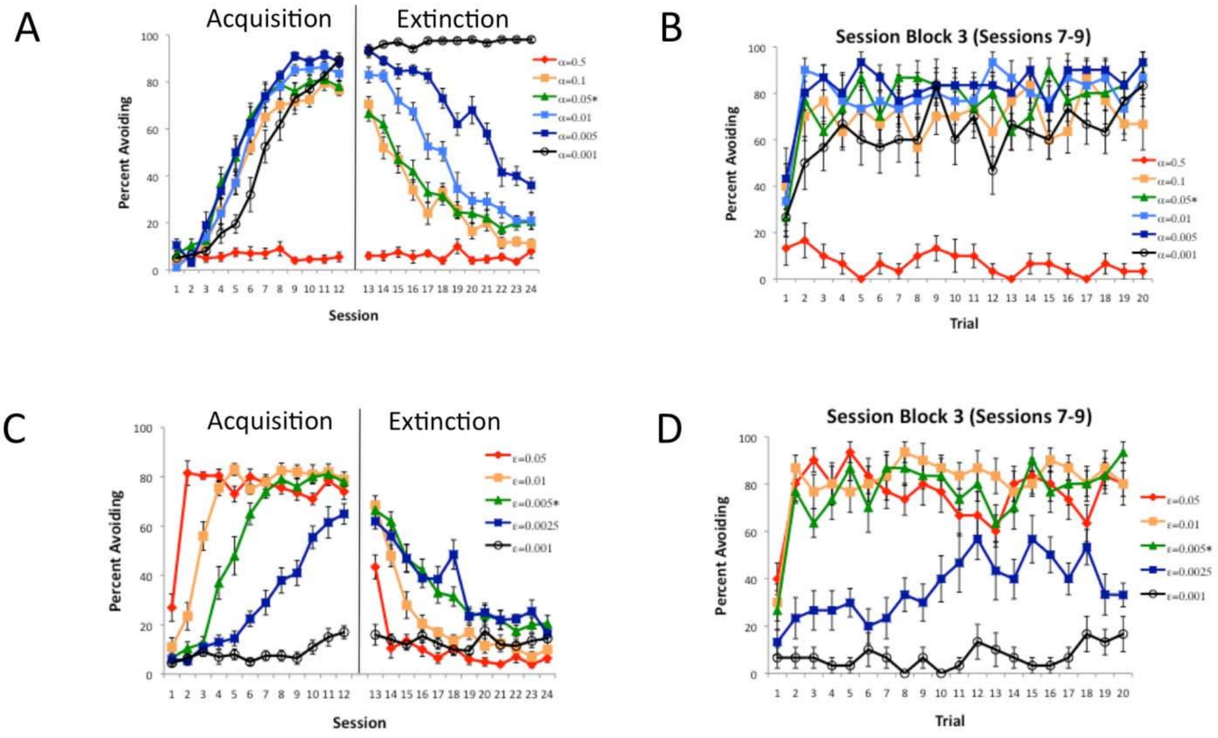


Figure S2.Parametric manipulations of the learning rates. Manipulating α, the learning rate in the critic module, has relatively little effect on (A) acquisition or (B) warm-up, except as values get very high (e.g. α=0.5); however, smaller values of α impair extinction (A). High and low values of ε, the learning rate in the actor module, produce correspondingly fast and slow rates of acquisition and extinction (C); for values of ε under which acquisition occurs, warm-up is preserved (D).

The parameter ε governs the rate at which the actor updates its weights, affecting the ability of particular inputs to evoke particular responses. Unsurprisingly, this parameter profoundly affects

both acquisition and extinction, although there is less effect on warm-up. For an intermediate value near 0.005, smooth acquisition and extinction curves are obtained (Figure S2C), but higher values produce extremely rapid acquisition and extinction, and lower values greatly retard acquisition. Interestingly, even the largest values of $\varepsilon$ explored, which produce very rapid acquisition and extinction (within only 2-4 sessions), do not eliminate the warm-up effect (Figure S2D). An intermediate value of $\varepsilon$=0.005 produces learning and extinction curves similar to those seen in the SD rat, and was used in the SD simulations.

### Shock intensity, $R_{shock}$

Finally, learning is also affected by the value of $R_{shock}$, the punishing value of shock. Figure S3A shows that acquisition is faster as $R_{shock}$ increases in magnitude. As would be expected, changes in $R_{shock}$ have relatively little effect on extinction (since shock is not presented); there is also little effect on warm-up (Figure S3B). $R_{shock}$ was set to the intermediate value of -4 in the SD simulations.

Figure S3

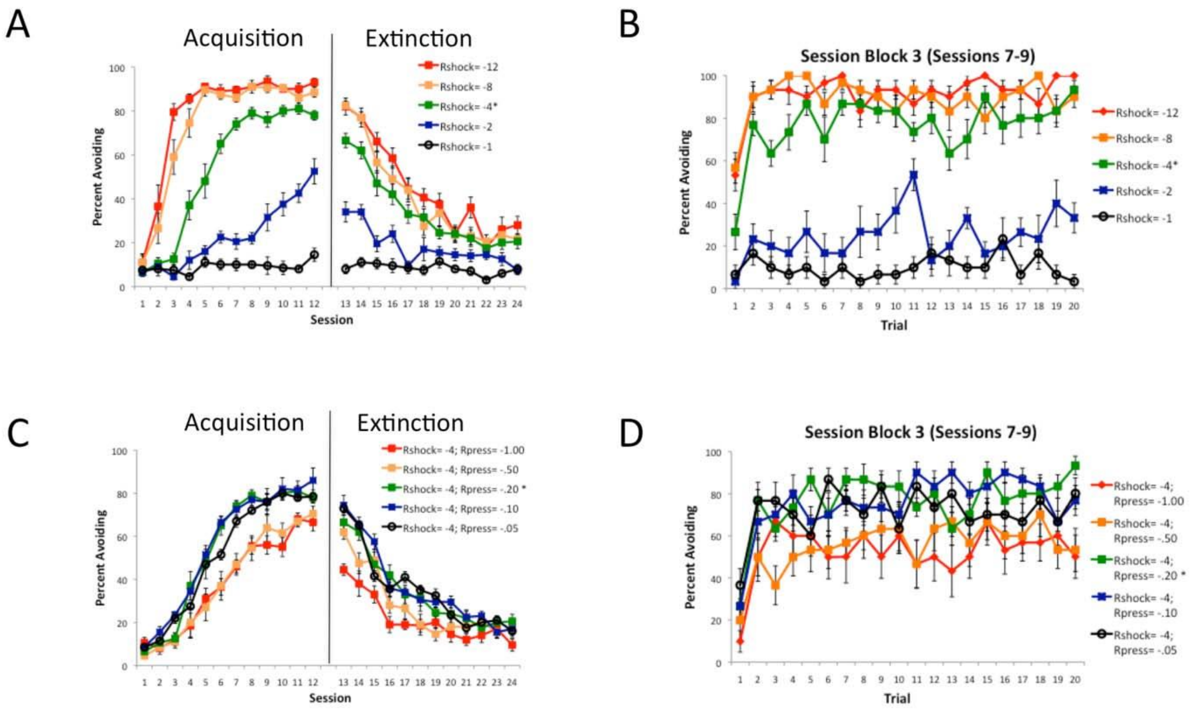


Figure S3.Parametric manipulations of the punishing value of shock, $R_{shock}$, and of leverpress, $R_{press}$. (A) Unsurprisingly, acquisition is faster, and extinction slower, as $R_{shock}$ increases; (B) for values of $R_{shock}$ under which acquisition occurs, warm-up is preserved (see also Figure 5, SD curves). (C,D) Given an intermediate value of $R_{shock}$= -4, there is relatively little effect of varying the reinforcement value of leverpress, $R_{press}$, as long as $R_{press}$ is small relative to $R_{shock}$.

As important as the value of $R_{shock}$ is the ratio between $R_{shock}$ and $R_{press}$, the punishing value of leverpress. Figure S3C shows that, as long as $R_{press}$ is very small relative to $R_{shock}$, acquisition is fast, but as $R_{press}$ approaches $R_{shock}$, and leverpress becomes almost as aversive as the shock itself, avoidance acquisition begins to degrade. Figure S3D shows that warm-up is not affected, even as $R_{press}$ approaches $R_{shock}$. $R_{press}$ was set to the value of -0.2 (i.e., 1/20$^{th}$ the value of $R_{shock}$) in the SD simulations reported here.
